# An analysis of information segregation in parallel streams of a multi-stream convolutional neural network

**DOI:** 10.1038/s41598-024-59930-7

**Published:** 2024-04-20

**Authors:** Hiroshi Tamura

**Affiliations:** 1 Cognitive Neuroscience Group, Graduate School of Frontier Biosciences, The University of Osaka, 1-4 Yamadaoka, Suita, Osaka 565-0871 Japan; 2 Center for Information and Neural Networks, Suita, Osaka 565-0871 Japan

**Keywords:** Neuroscience, Psychology

## Abstract

Visual information is processed in hierarchically organized parallel streams in the primate brain. In the present study, information segregation in parallel streams was examined by constructing a convolutional neural network with parallel architecture in all of the convolutional layers. Although filter weights for convolution were initially set to random values, color information was segregated from shape information in most model instances after training. Deletion of the color-related stream decreased recognition accuracy of animate images, whereas deletion of the shape-related stream decreased recognition accuracy of both animate and inanimate images. The results suggest that properties of filters and functions of a stream are spontaneously segregated in parallel streams of neural networks.

## Introduction

In the cerebral cortex, visual information is processed in hierarchically organized parallel pathways^1–7^. In the lower visual cortical areas, such as the primary visual cortex (V1) and secondary visual area (V2), color information and orientation information are processed in different cortical modules^2,6,8–18^. In the higher visual cortical areas, color information and shape information are processed in a segregated manner^19–22^. Furthermore, animate images are processed in a segregated manner from inanimate images in the inferior temporal cortex^23–26^. Thus, parallel functional organization has been observed throughout the visual cortical areas. However, it remains unclear how this functional segregation emerges.

In the present study, information segregation in a parallelized convolutional neural network (CNN) was studied to explore the possibility of spontaneous segregation of visual information in parallel streams. CNNs are hierarchically organized feed-forward networks for classification of inputs, such as visual object images, and consist of multiple sets of layers, with each set of layers performing convolution, thresholding, and pooling^27^. Filter weights for convolution are acquired during training. As a result of training, the filter structure of the first convolution layer becomes similar to the receptive-field structure of simple cells of the primary visual cortex^27^. Pooling provides position invariance in CNNs, which is also observed in neurons of visual cortices^28^. Some similarities between CNN outputs and the activity of neurons in the primate visual cortices have been described in previous studies^29–34^.

**Figure 1:**
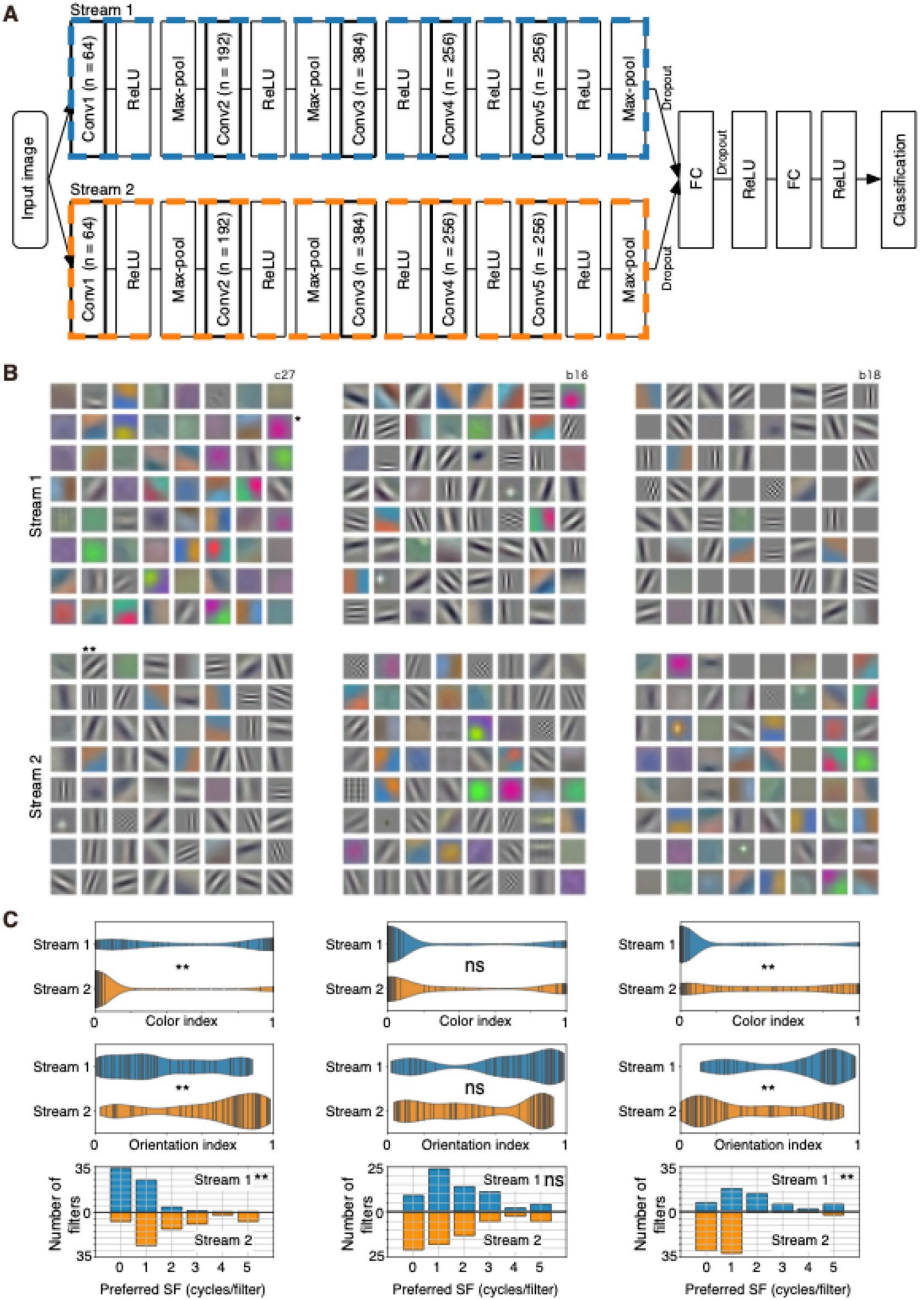
(**A**) Architecture of two-streams fully parallel (2SFP) AlexNet. Each stream of 2SFP-AlexNet contains five convolutional layers (conv1–5) with an activation function (ReLU), and three pooling layers (Max-pool). Outputs from two streams were combined and fed into fully connected (FC) layers and the output layer for classification. The number of filters in convolutional layers were indicated in parentheses. (**B**) Conv1-filters from stream 1 (top) and those from stream 2 (bottom) of three representative instances (left, center, right). For visualization, the minimum and maximum weight values were scaled between 0 and 255. Single and double asterisks are filters mentioned in the main text. (**C**) Comparisons of color index (top), orientation index (middle), and preferred spatial frequency (cycles/filter) of conv1 filters of stream 1 (blue) and stream 2 (orange) for the three instances in (**B**). In the violin plots, kernel density estimation was performed, and vertical bars show each underlying datapoint. Double asterisks indicate significant differences between two distributions (*p* < 0.01). “ns” indicates non-significant differences.

In the current study, I constructed a modified version of AlexNet^27^, called the two-streams fully parallel (2SFP) AlexNet (Fig. 1A). I introduced parallel architecture to AlexNet in all convolutional layers. This architecture allows a comparison of filter properties in both lower and higher layers, and analysis of the effect of deletion of a stream.

## Results

2SFP-AlexNet contains two streams of five hierarchically organized convolutional layers (conv1–5) and three pooling layers. Outputs from the two streams are concatenated and fed into fully connected layers, then to the output layer (Fig. 1A). The present study was based on 16 instances (Table 1) of 2SFP-AlexNet. Each model instance was trained with randomly initialized parameters and with some variation in the initial learning rate and batch size (Table 1). In addition, one instance of 2SFP-VGG11, one instance of 2SFP-ResNet26, and one instance of three-streams fully parallelized (3SFP) AlexNet were also examined (Table 1). These networks were trained for classification of 1000 object-image categories using the ImageNet database^35^. After training, top-5 accuracy of 2SFP-AlexNet was 0.484–0.532 with the validation set. Although the performance was lower than that of the original AlexNet model, filters were well trained and matured for the present purpose.

**Table 1:** Model instances.

Instances	Architecture	Number of streams	Batch size	Initial learning rate
a31	2SFP-AlexNet	2	128	0.01
b02	2SFP-AlexNet	2	128	0.01
b16	2SFP-AlexNet	2	128	0.01
c30	2SFP-AlexNet	2	128	0.01
101	2SFP-AlexNet	2	128	0.01
b25	2SFP-AlexNet	2	16	0.01
b18	2SFP-AlexNet	2	32	0.01
b30	2SFP-AlexNet	2	32	0.01
c12	2SFP-AlexNet	2	32	0.01
c19	2SFP-AlexNet	2	32	0.01
c21	2SFP-AlexNet	2	32	0.01
b28	2SFP-AlexNet	2	512	0.01
c23	2SFP-AlexNet	2	128	0.02
c27	2SFP-AlexNet	2	128	0.02
c28	2SFP-AlexNet	2	128	0.005
105	2SFP-AlexNet	2	128	0.005
405	2SFP-VGG11	2	32	0.01
B15	2SFP-ResNet26	2	32	0.01
b21	3SFP-AlexNet	3	32	0.01

### Properties of filters in convolutional layer 1 of two-streams fully parallelized AlexNet

After training, conv1 filters acquired a variety of kernels. Properties of conv1 filters were examined by visualizing and analyzing their input weights. Some filters were color selective while others were orientation selective (Fig. 1B), and some filters preferred lower spatial frequencies while others preferred higher spatial frequencies. A conv1 filter (rightmost filter in the second row of Fig. 1B-top left, *) of stream 1 of a model instance preferred red color, showed no orientation selectivity, and preferred lower spatial frequencies. The color index and orientation index values of the filter were 0.994 and 0.0273, respectively, and the preferred spatial frequency (SF) was 0 (i.e., direct current [DC]). A conv1 filter of stream 2 (second filter in the top row of Fig. 1B-bottom left, **) of the same model instance showed no color selectivity (color index, 0.00102) but preferred an oblique orientation (orientation index, 0.770) and preferred a middle spatial frequency (preferred SF, 2 cycles/filter).

**Figure 2:**
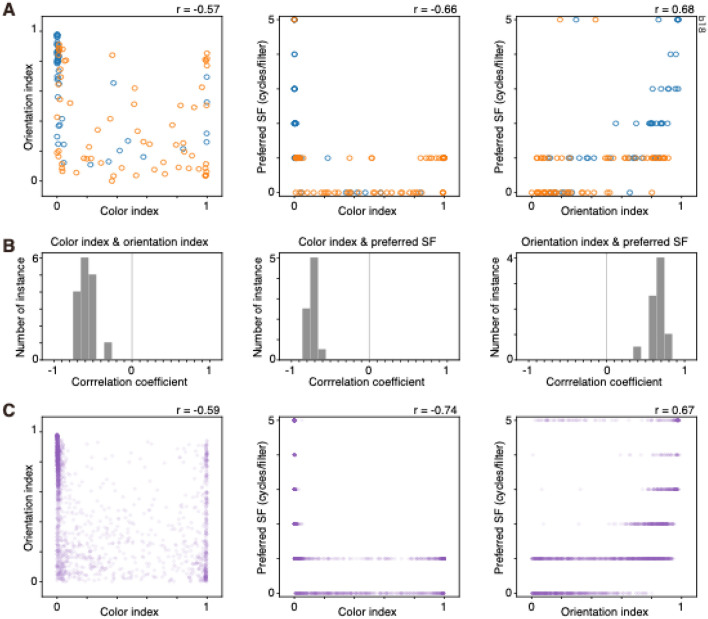
Relationships among color index, orientation index and preferred spatial frequency (SF) of conv1 filters of two-streams fully parallel AlexNet. (**A**) Relationships between color index and orientation index (left), between color index and preferred SF (center), and between orientation index and preferred SF (right) in a model instance. This instance is the same as that shown in Fig. 1-right. Each circle represents a filter of conv1 of a stream 1 (blue) or stream 2 (orange). Correlation coefficient (*r*) is provided for each panel. (**B**) Frequency distributions of correlation coefficient between color index and orientation index (left), color index and preferred SF (center), and orientation index and preferred SF (right) from 16 model instances. (**C**) Relationships between color index and orientation index (left), between color index and preferred SF (center), and between orientation index and preferred SF (right) with all of the conv1 filters from 16 model instances. The number of filters plotted was 1895. Each circle represents a filter of conv1. Correlation coefficient (*r*) is provided for each panel.

To examine whether color information and orientation information are encoded by different filters, the relationship between color index and orientation index was examined. If each filter is dedicated to a single function, such as color or orientation, color-selective filters are less orientation selective and orientation-selective filters are less color selective. In this case, a negative relationship between color index and orientation index was expected. The degree of color selectivity, degree of orientation selectivity, and preferred SF were related to each other. In the instance shown in Fig. 1B-right, the color index was negatively correlated with the orientation index (*r* = − 0.57, n = 117 [11 filters with flat kernel were excluded from the analysis], Spearman’s rank correlation; Fig. 2A-left) and with preferred SF (*r* = − 0.66; Fig. 2A-center), and the orientation index was positively correlated with preferred SF (*r* = 0.68; Fig. 2A-right). These relationships were consistently observed in all 16 instances (Fig. 2B,C). These results suggested that color information and orientation information were encoded by different populations of filters, and color-selective filters were less orientation selective and tended to prefer lower SF, while orientation-selective filters were less color selective and preferred higher SF. Although the color index was negatively correlated with the orientation index, there was a small but significant fraction of filters that simultaneously had a higher color index as well as a higher orientation index (Fig. 2A,C, left panel, points around the upper right corner; see for example, the 42nd filter, 2nd filter in the 6th row of stream 2 of Fig. 1B-right, horizontally oriented yellow and blue filter), suggesting that some filters were selective to both color and orientation^13,17^.

The results described above raise the question of how these conv1 filters are associated with the two streams of 2SFP-AlexNet. I found that color-selective filters were numerous in a stream, and orientation-selective filters were numerous in the other stream in most instances. As a result, selectivity indices and preferred SF of conv1 filters differed between the two streams of 2SFP-AlexNet. For example, the median color index values of stream 1 and 2 of Fig. 1B-left were 0.46 and 0.0050, respectively, and the median orientation index values of stream 1 and 2 were 0.33 and 0.77, respectively. These indices differed between streams (color index, *p* = 4.93 × 10^−12^; orientation index, *p* = 3.58 × 10^−6^; Mann–Whitney U test; Fig. 1C-left). Preferred SF also differed between the two streams (*p* = 9.05 × 10^−10^; Fig. 1C-left). The mean preferred SFs of stream 1 and 2 were 0.56 and 1.91, respectively. As a result, in the instance shown in Fig. 1B-left, conv1 filters in stream 1 had a higher degree of color selectivity, a lower degree of orientation selectivity, and lower preferred SF compared with those in the other stream.

**Figure 3:**
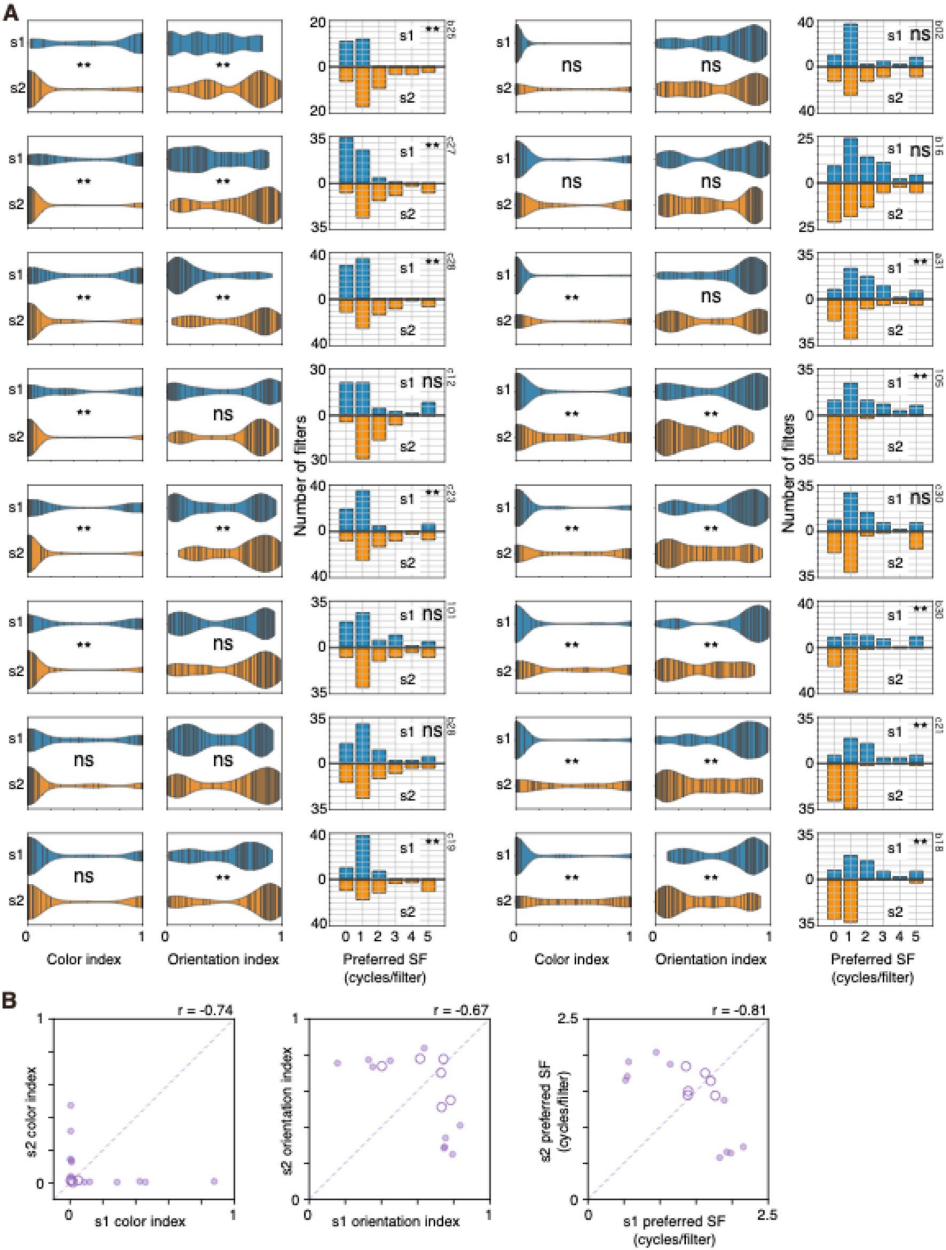
(**A**) Comparisons of color index (top), orientation index (middle), and preferred spatial frequency (SF, cycles/filter) of conv1 filters of stream 1 (s1, blue) and stream 2 (s2, orange) of two-streams fully parallel AlexNet for the 16 instances. (**B**) Comparisons of the median color index (left) and the median orientation index (center) and the mean preferred SF (right) of conv1 filters of stream 1 (s1, horizontal axis) and stream 2 (s2, vertical axis) across 16 instances. Closed and open circles show the significant and non-significant differences between the distributions (*p* < 0.01), respectively. The diagonal broken line is the equality line. Correlation coefficient (*r*) is provided for each panel. Other conventions are as in Fig. 1.

Significant differences in color index values, orientation index values, and preferred SF were also observed in the instance shown in Fig. 1B-right. In this instance, conv1 filters in stream 1 had a lower degree of color selectivity, a higher degree of orientation selectivity and preferred higher SF compared with those in the other stream (Fig. 1C-right). Among the 16 instances of 2SFP-AlexNet, differences in the color index and orientation index were observed in 12 and 10 instances, respectively (Fig. 3A). Differences in preferred SF were observed in 10 instances (Fig. 3A). Differences in color index values, orientation index values, and preferred SF were simultaneously observed in eight instances (Fig. 3A). In all eight instances, a stream tended to have conv1 filters with strong color selectivity, weak orientation selectivity, and a preference for lower SF, and the other stream tended to have conv1 filters with weak color selectivity, strong orientation selectivity, and a preference for higher SF. In Fig. 3B, the median color index, median orientation index, and mean preferred SF of conv1 filters of stream1 were plotted against those of stream 2. In general, if a median index of a stream was high, the median index of the other stream was low, and there was a negative correlation between streams (Fig. 3B left: color index, − 0.74; middle: orientation index, − 0.67; right: preferred SF, − 0.81), also suggesting the segregation of filter properties between streams.

However, such segregation was not observed in all model instances. For example, in the instance shown in Fig. 1B-center, the median color index values of conv1 filters of stream 1 and 2 were 0.0068 and 0.019, respectively, and the index did not differ between the streams (*p* = 0.188; Fig. 1C-center). The median orientation index of conv1 filters of stream 1 and 2 were 0.73 and 0.51, respectively, and the index did not differ between the streams (*p* = 0.030; Fig. 1C-center). The mean preferred SF of conv1 filters of stream 1 and 2 were 1.77 and 1.44, respectively, and preferred SF also did not differ between the streams (*p* = 0.073; Fig. 1C-center). Furthermore, even in the instance with a significant difference in indices, the degree of difference in indices and preferred SF varied across instances. The plots shown in Fig. 3B revealed that some points were closer to the equality line while others were further away from it, suggesting that a degree of segregation varied among instances. Such a degree of segregation could potentially be related to hyperparameters of AlexNet (see Table 1). Indeed, there was a tendency for small batch size to cause a higher degree of segregation. However, even among instances with the same batch size, there were substantial variations in the degree of segregation.

**Figure 4:**
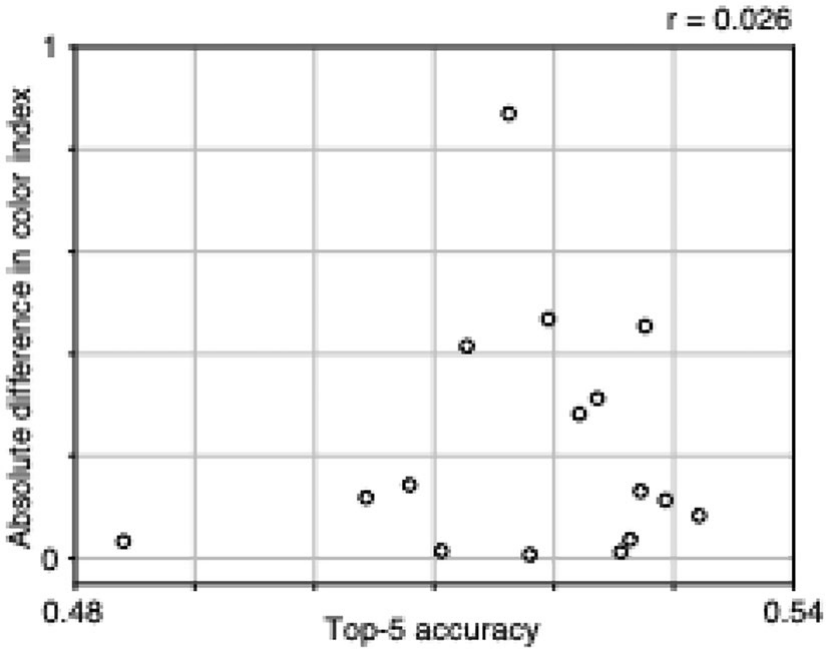
Relationships between the top-5 accuracy and the absolute difference between streams in the median color index of conv1 filters. Each point represents a model instance. Correlation coefficient (*r*) is provided.

The degree of segregation of filter properties was not related to the image-classification performance of 2SFP-AlexNet. In the Fig. 4, top-5 accuracy was plotted on the horizontal axis, and absolute differences in color index was plotted on the vertical axis. The correlation coefficient between the two values was 0.026 (Spearman’s rank correlation), suggesting independence.

### Properties of filters in convolutional layer 2–5 of two-streams fully parallelized AlexNet

**Figure 5:**
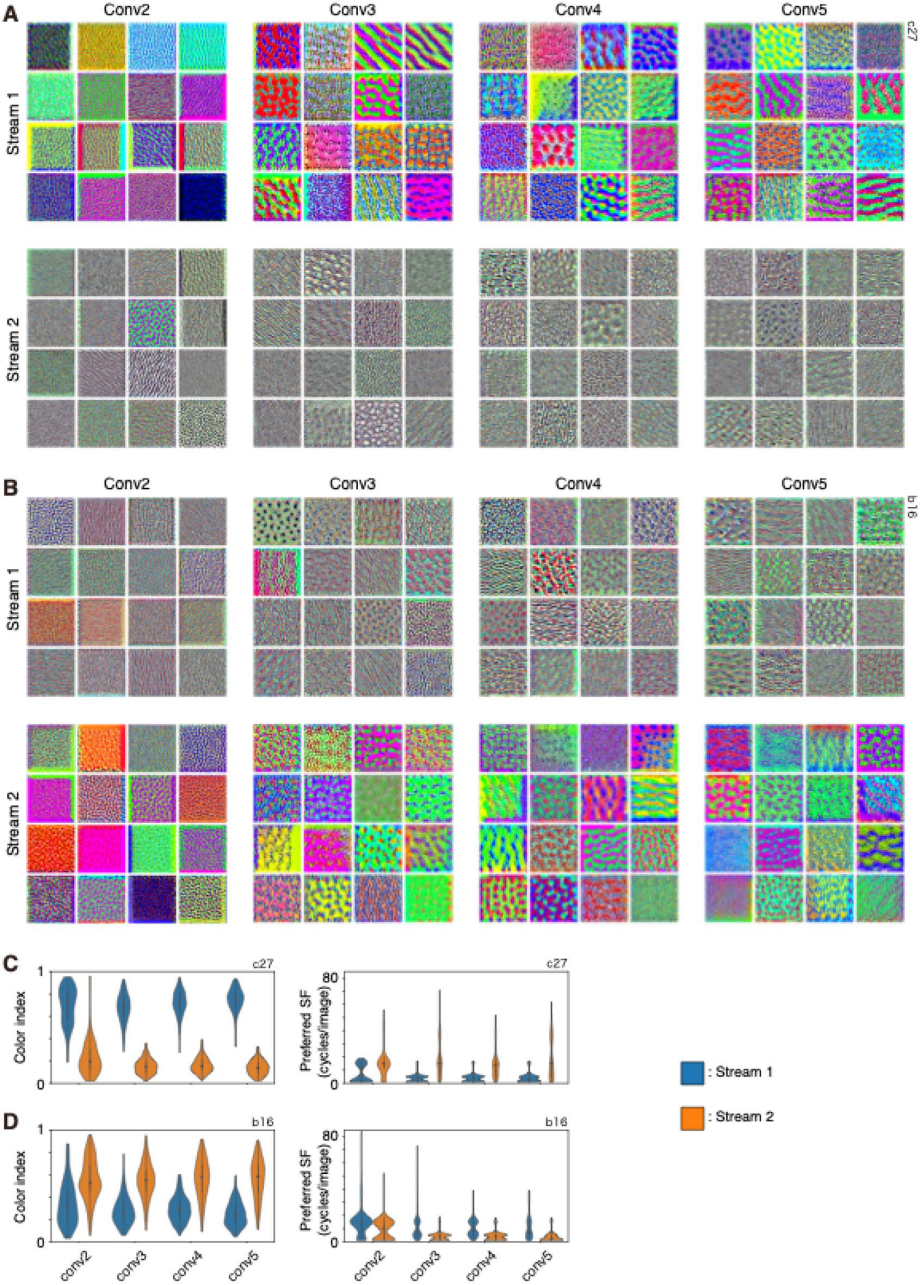
Comparisons of most effective stimuli (MESs) of conv2–5 filters of stream 1 and stream 2 of two-streams fully parallel AlexNet. (**A**,**B**) Sixteen examples of MESs each from conv2–5 of stream 1 (top) and stream 2 (bottom) of a model instance (**A**) and another instance (**B**). (**C**,**D**) Comparisons of color index and preferred SF of conv2–5 filters of stream 1 (blue) and stream 2 (orange) of A (**C**) and B (**D**). Other conventions are as in Fig. 1.

**Figure 6:**
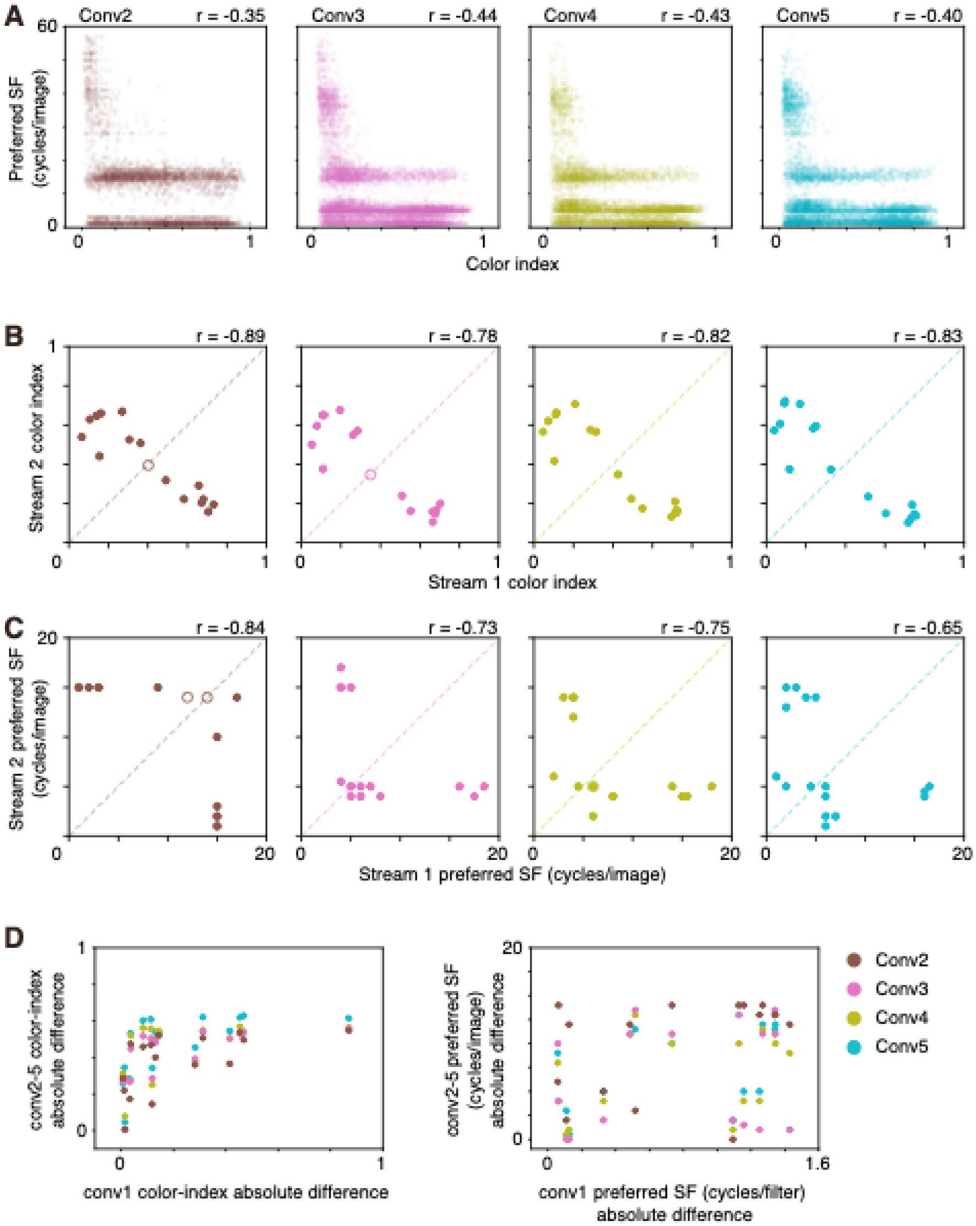
Relationships between color index and preferred spatial frequency (SF) of most effective stimuli (MESs) of conv2–5 filters of two-streams fully parallel AlexNet. (**A**) Relationships between color index and preferred SF (cycles/image) of MESs of all of the filters from all 16 model instances of conv2 (left), conv3 (center-left), conv4 (center-right), and conv5 (right). Each point represents single filter. Correlation coefficient (*r*) is provided for each panel. (**B**,**C**) Comparisons of the median color index (**B**) and the median preferred SF (**C**) of MESs of conv2 (left), conv3 (center-left), conv4 (center-right) and conv5 (right) filters of stream 1 (horizontal axis) and stream 2 (vertical axis) across 16 instances. Correlation coefficient (*r*) is provided for each panel. (**D**), Relationships between the absolute difference between streams in the median color index of conv1 filters and that of MESs of conv2–5 filters (left). Relationships between the absolute difference between streams in the mean preferred SF of conv1 filters and that in the median preferred SF of MESs of conv2–5 filters (right). Each point represents a model instance, with brown for conv2 filters, pink for conv3 filters, olive for conv4 filters, and cyan for conv5 filters.

To examine the properties of filters in higher convolutional layers, the stimulus image that induced the greatest activation in each filter (most effective stimulus [MES]) was calculated using gradient ascent starting from an initial image with random RGB values^36,37^. MESs for some filters of conv2–5 were colorful, whereas those for other filters were colorless (see Fig. 5A,B). The higher SF component was stronger in some MESs, whereas the lower SF component was stronger in some other MESs. Degree of color selectivity and preferred SF of MESs were related to each other in conv2–5, and color selective MESs tended to contain a lower SF component, whereas color-non-selective MESs contained a variety of SFs. The results revealed that color index values were negatively correlated with preferred SF in conv2–5 (*r* = − 0.44 to − 0.35, Spearman’s rank correlation; Fig. 6A).

Filter properties of conv2–5 examined using MES differed between streams. In the instance shown in Fig. 5A, the color index of MESs of conv2–5 of stream 1 (0.68–0.76, median) was larger than that of stream 2 (0.14–0.19; *p* = 3.78 × 10^−56^–6.10 × 10^−127^, Mann–Whitney U test; Fig. 5C-left). Preferred SF of MESs also differed between the two streams (*p* = 1.87 × 10^−14^–1.21 × 10^−80^; Fig. 5C-right) and preferred SF of conv2–5 of stream 1 (2–4, median) was lower than that of stream 2 (14–15, median). In another instance shown in Fig. 5B, the color index of MESs of conv2–5 differed between the two streams (*p* = 4.57 × 10^−23^–2.66 × 10^−83^; Fig. 5D-left). Preferred SF of MESs also differed between the two streams (*p* = 3.16 × 10^−8^–6.79 × 10^−45^; Fig. 5D-right). Significant differences in color index values of MESs of conv2, 3, 4, and 5 were observed in 15, 15, 16, and 16 instances, respectively (Fig. 6B). A significant difference in preferred SF of MESs of conv2, 3, 4, and 5 was observed in 14, 16, 15, and 16 instances, respectively (Fig. 6C). The median color index value of a stream was negatively correlated with that of the other stream (− 0.78 to − 0.89; Fig. 6B), and the median preferred SF of a stream was also negatively correlated with that of the other stream (− 0.65 to − 0.84; Fig. 6C). Thus, color-selective filters that preferred lower SF in conv2–5 were segregated from color-non-selective filters that preferred higher SF in conv2–5. The plots also revealed that some points were closer to the equality line while others were further away from it, suggesting that the degree of segregation varied among instances.

In the instance shown in Fig. 5A, MESs of filters of conv2–5 in stream 1 appear colorful, while those in stream 2 appear colorless. These properties may be derived from the properties of conv1 filters, because color-selective filters were concentrated in conv1 of stream 1 and colorless filters were concentrated in conv1 of stream 2 of the instance (see Fig. 1B-left). However, in another instance of Fig. 5B, where color-selective filters were observed in both conv1 of stream 1 and stream 2 (see Fig. 1B-center) and color index values did not differ between streams, MESs of filters of conv2–5 in stream 2 appeared to be more colorful than those of stream 1. To examine whether the difference in color selectivity and SF preference of conv2–5 was inherited from those of conv1, the correlation between the absolute difference in color index of conv1 filters and that of conv2–5 was examined. There was a positive correlation between these measures in conv2–5 (*r* = 0.61–0.80, Spearman’s rank correlation; Fig. 6D-left), suggesting that the difference in color index of MESs of filters of conv2–5 is likely to be inherited from the difference observed in conv1. In contrast, the correlation between the absolute difference in preferred SF of conv1 filters and that of conv2–5 was weak (*r* = 0.21–0.41, Spearman’s rank correlation; Fig. 6D-right). These results suggest that the difference in color selectivity was inherited from that of conv1, while this tendency was weak for SF preference.

### A comparison of stimulus representation between two-streams fully parallelized AlexNet

**Figure 7:**
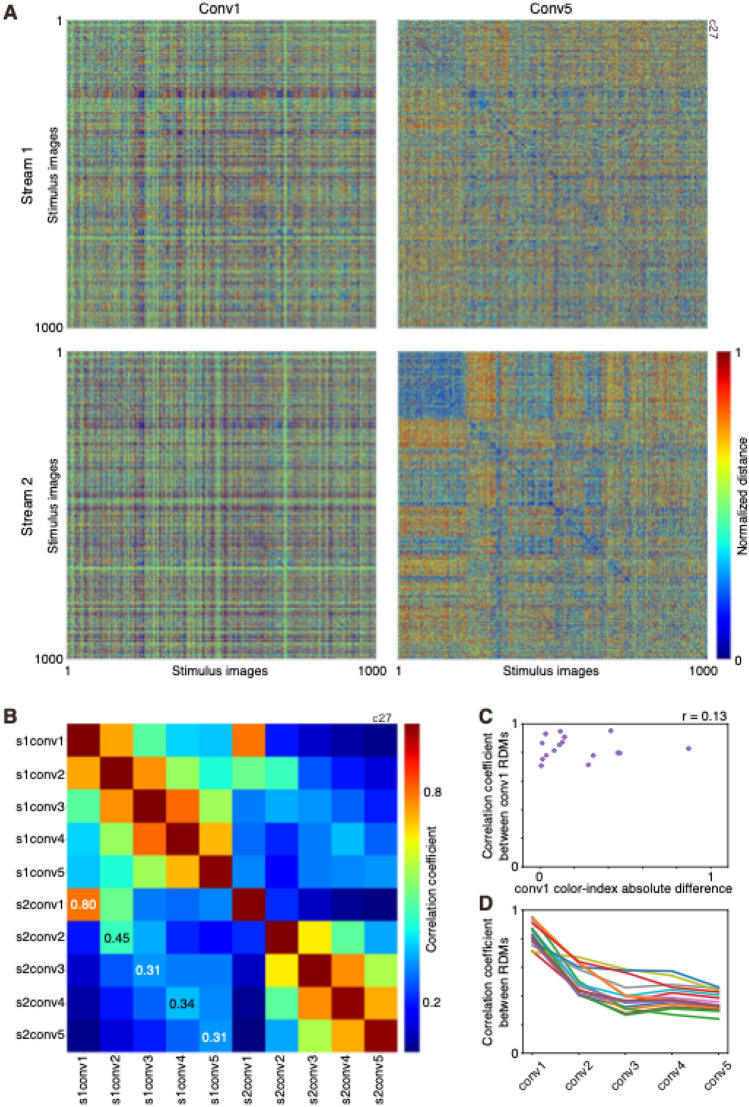
Comparisons of representational dissimilarity matrix (RDM) between streams of two-streams fully parallel (2SFP) AlexNet. (**A**) RDMs of a model instance calculated with the outputs from conv1 (left column) and conv5 (right column) filters of stream 1 (top row) and stream 2 (bottom row) to 1000 stimulus images. Distance between stimulus images were rank ordered and normalized between 0 and 1 and plotted in a color scale. (**B**) Correlation coefficient between RDMs between conv layers. Correlation coefficients were color coded. The correlation coefficient between the same layer (the diagonal element) was the mean across 10 correlation coefficients, each of which was calculated by randomly dividing filters into two groups. The five numbers on the plot are correlation coefficients between streams at the same hierarchical level. (**C**) Relationship between absolute difference between streams in the median color index of conv1 filters and correlation coefficient of RDMs of conv1 filters. Each point corresponds to a single model instance. Correlation coefficient (*r*) is provided. (**D**) Changes in correlation coefficients of RDMs along the hierarchy of 2SFP-AlexNet. Each line corresponds to a single model instance.

To examine how the difference in filter properties contributes to the difference in information representation between streams, I compared the representation of a set of 1000 stimulus images between streams of 2SFP-AlexNet by calculating the representational dissimilarity matrix (RDM)^24^ (Fig. 7A). Each pixel in the RDM represents rank ordered and normalized distance between paired stimulus images calculated with outputs of a set of filters to the stimulus images. The RDM of conv1 of stream1 was similar to that of stream 2 (Fig. 7A-left) despite the difference in the degree of color and orientation selectivity and preferred SF (see Fig. 1B-left). Similarity in stimulus representation was quantified by calculating the correlation coefficient between the RDMs (Fig. 7B). In the instance of Fig. 7A, the correlation coefficient of the RDM of conv1 between the two streams was 0.80 (Fig. 7B). Note that large differences in color index, orientation index, and SF preference of conv1 filters between streams were observed in the instance shown in Fig. 7A and B (see Fig. 1B-left). This result suggests that similarity in image representation of conv1 filters between streams was not related to similarity in the degree of color and orientation selectivity and SF preference. Indeed, the correlation coefficients of the RDM of conv1 filters between streams obtained with all 16 instances were always high (0.71–0.95) and were not related to the absolute difference in color index between streams (*r* = 0.13, Spearman’s rank correlation; Fig. 7C).

Contrary to RDM of conv1, RDM of conv5 of stream1 was different from that of stream 2 (Fig. 7A-right). In the instance shown in Fig. 7A, the correlation coefficient of RDM of conv5 between the two streams was 0.31 (Fig. 7B). Thus, the correlation coefficients between RDMs from different streams at the same hierarchical level decreased gradually along the hierarchy of 2SFP-AlexNet. This tendency was confirmed for all 16 instances (Fig. 7D). The correlation coefficient of RDMs differed among conv layers (*p* = 1.40 × 10^−11^, Friedman test for repeated samples), and the correlation coefficient of RDMs of conv5 (0.33, median) was smaller than that of conv1 (0.82). This result suggests that similarity in information representations between two streams decreased during hierarchical processing from conv1 to conv5 and representations became less correlated between streams.

### Effects of deletion of a stream of two-streams fully parallelized AlexNet on the classification of images

**Figure 8:**
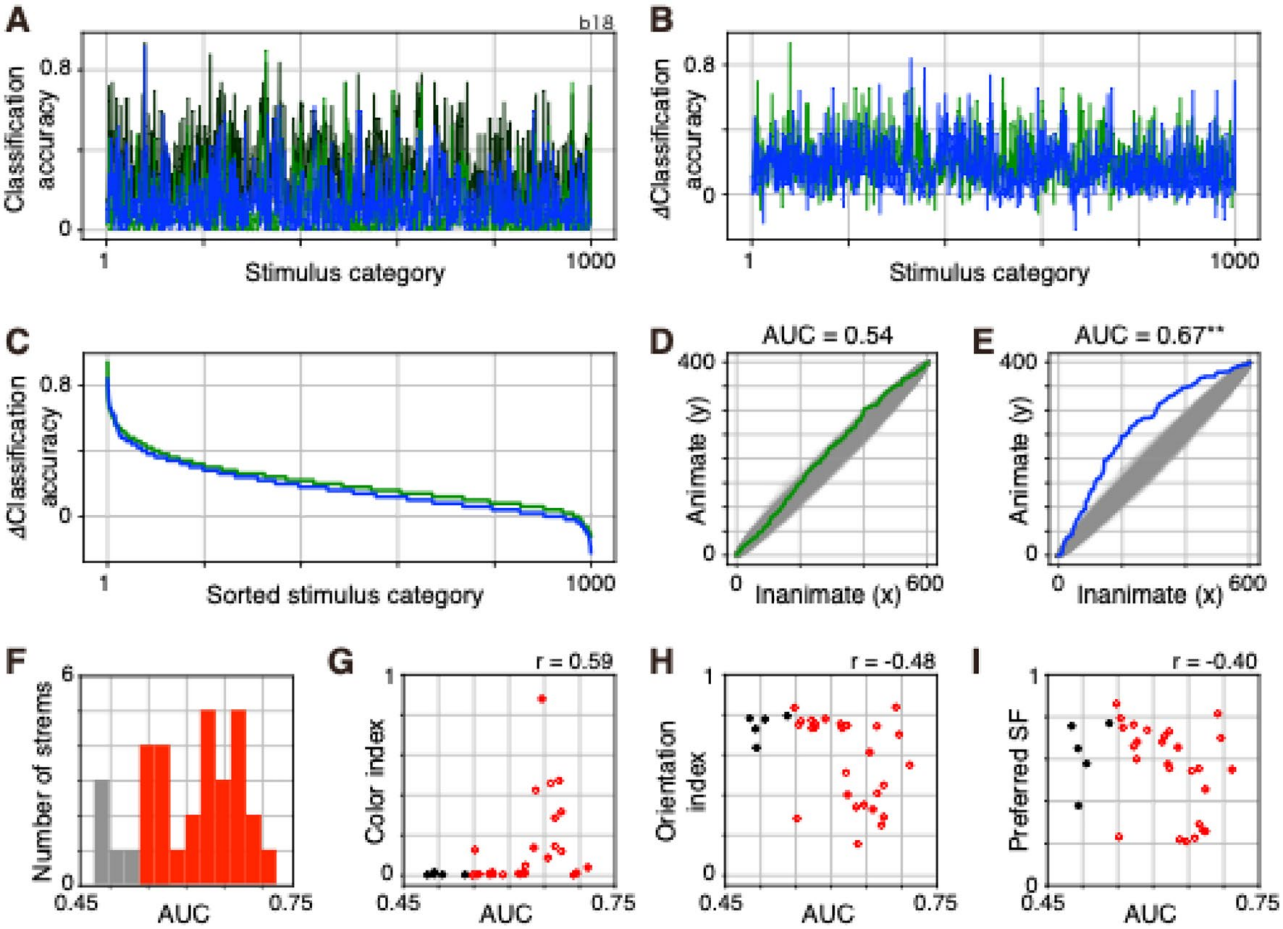
Effect of deletion of a stream of two-streams fully parallel (2SFP) AlexNet on the classification accuracy of image categories. (**A**) Classification accuracy (top-1 accuracy) of 1000 image categories for original 2SFP-AlexNet (black), stream-1 deleted 2SFP-AlexNet (green), and stream-2 deleted 2SFP-AlexNet (blue). (**B**) Difference in classification accuracy (ΔClassification accuracy) between original 2SFP-AlexNet and stream-1 deleted 2SFP-AlexNet (green), and that between original 2SFP-AlexNet and stream-2 deleted 2SFP-AlexNet (blue). (**C**) The same plot as in (**B**), but sorted in descending order. (**D**,**E**) Two-dimensional (2D) plots of the descending order of the animate and inanimate super-categories and area under the curve (AUC) analysis. 2D plot of the difference between original 2SFP-AlexNet and stream-1 deleted 2SFP-AlexNet (**D**, green) and that between original 2SFP-AlexNet and stream-2 deleted 2SFP-AlexNet (**E**, blue). Results obtained with label (animate or inanimate) randomized data were also provided (gray). Normalized AUC values are provided for each graph. Double asterisk indicated a significant difference between the AUC and that calculated using label randomized data. (**F**) Frequency distribution of significant (red) and non-significant (gray) AUCs. Thirty-two streams from 16 model instances were plotted. (**G**–**I**) Relationships between AUC and color index of conv1 filters (**G**), AUC and orientation index of conv1 filters (**H**), and AUC and preferred spatial frequency of conv1 filters (**I**). Red open circles represent significant AUCs. Black filled circles represent non-significant AUCs. Correlation coefficient (*r*) is provided for each panel.

If each of the two streams of 2SFP-AlexNet represents images in a different manner, the effect of deleting one stream on classification accuracy is likely to be different from that of deleting the other stream. To examine the contribution of each stream to image classification, a deletion experiment was performed. To delete a stream, output values of the last max-pool layer of the stream was forced to be set to zero. The correct proportion was calculated with the validation set for original 2SFP-AlexNet, stream-1 deleted 2SFP-AlexNet, and stream-2 deleted 2SFP-AlexNet. Because the relationships between the effects of deletions and animate and inanimate super-categories emerged in a pilot analysis, each of the 1000 image categories was classified into animate or inanimate super-categories. The animate super-category included 398 image categories and the inanimate super-category included 602 image categories. Classification accuracy (top-1 accuracy) for the 1000 image categories was calculated for original 2SFP-AlexNet, stream-1 deleted 2SFP-AlexNet, and stream-2 deleted 2SFP-AlexNet (Fig. 8A). Next, differences in classification accuracy (ΔClassification accuracy = accuracy of original 2SFP-AlexNet − accuracy of stream deleted 2SFP-AlexNet) between original 2SFP-AlexNet and stream-1 deleted 2SFP-AlexNet, and between original 2SFP-AlexNet and stream-2 deleted 2SFP-AlexNet were calculated by subtracting the classification accuracy of stream deleted 2SFP-AlexNet from that of original 2SFP-AlexNet (Fig. 8B). The larger the ΔClassification accuracy, the stronger the effect of stream deletion. ΔClassification accuracy was sorted in descending order (Fig. 8C). Because each image category was labeled as the animate or inanimate super-category, the rank order of animate and inanimate super-categories was obtained. Finally, the descending order of animate and inanimate categories was plotted in an x–y plane (Fig. 8D,E; see Supplementary Fig. 1 for the details of the method). The area under the curve (AUC) of the plot of Fig. 8D and E was calculated. AUC was normalized with the product of the number of image categories in the animate super-category and that in the inanimate super-category. AUC takes a value between 0 and 1. AUC would be larger than 0.5 if many image categories labeled as animate super-category showed large decreases in the proportion of correct responses and had higher ranks than those labeled as inanimate super-category. AUC would be smaller than 0.5 if many image categories labeled as inanimate super-category showed large decreases in the proportion of correct responses and had higher ranks than those labeled as animate super-category. Thus, AUC captures the difference in the effect of stream deletion on classification accuracy between the animate and inanimate super-categories. Significance of AUC was examined by comparing the value with AUCs calculated using label (animate or inanimate) randomized data (*number of randomizations* = 1000).

By deleting stream 1 of the instance in Fig. 8A, top-5 accuracy was decreased from 0.52 to 0.22. AUC was 0.54 and was no different from AUCs obtained with label randomized data (*p* = 0.032; Fig. 8D), suggesting that stream 1 was related to classification of image categories in both animate and inanimate super-categories. By deleting stream 2 of the instance in Fig. 8A, top-5 accuracy was decreased from 0.52 to 0.29. AUC was 0.67, which was larger than AUCs obtained with label randomized data (*p* < 0.001; Fig. 8E), suggesting that stream 2 was related to classification of image categories of the animate super-category.

In 27 of the 32 streams from 16 instances of 2SFP-AlexNet, deletion resulted in a significant difference (*p* < 0.01) in AUC (Fig. 8F). In all of the significant cases, AUCs were larger than 0.5, meaning that deletion of a stream decreased classification performance of image categories in the animate super-category. In 13 of the 16 instances of 2SFP-AlexNet, deletion resulted in a significant difference (*p* < 0.01) in AUC between two streams, and AUCs of each stream were negatively correlated with those of the other stream (*r* = − 0.91), suggesting that if a stream contributed more to the classification performance of image categories in the animate super-category, the contributions of the other stream to that super-category were relatively small.

In the above analysis, deletion was performed by replacing the output from a stream with zero. Here, deletion was performed by replacing the output from a stream with its average output value, thus keeping the overall activity level but eliminating the variance in output values across units. Two sets of AUC were obtained with different deletion methods; one method replaced the output with zero and the other replaced the output with its average. The two sets were correlated with each other (*r* = 0.962, Spearman’s rank correlation), suggesting that overall activity level was not important. Instead, the analysis suggested the importance of variance in output values across units for classification performance.

To examine whether the degree of contributions to classification of image categories of the animate super-category was related to a specific type of filter property of conv1 layers, the relationships between AUC and selectivity indices of conv1 filters were examined. AUC was positively correlated with color index (*r* = 0.59; Fig. 8G), and was negatively correlated with orientation index (*r* = − 0.48; Fig. 8H). AUC was not correlated with preferred SF (*r* = − 0.40; Fig. 8I). The results suggested that animate super-category-related information was encoded by color-selective but less orientation-selective conv1 filters.

### Properties of two-streams fully parallelized VGG11, ResNet26 and three-streams fully parallelized AlexNet

**Figure 9:**
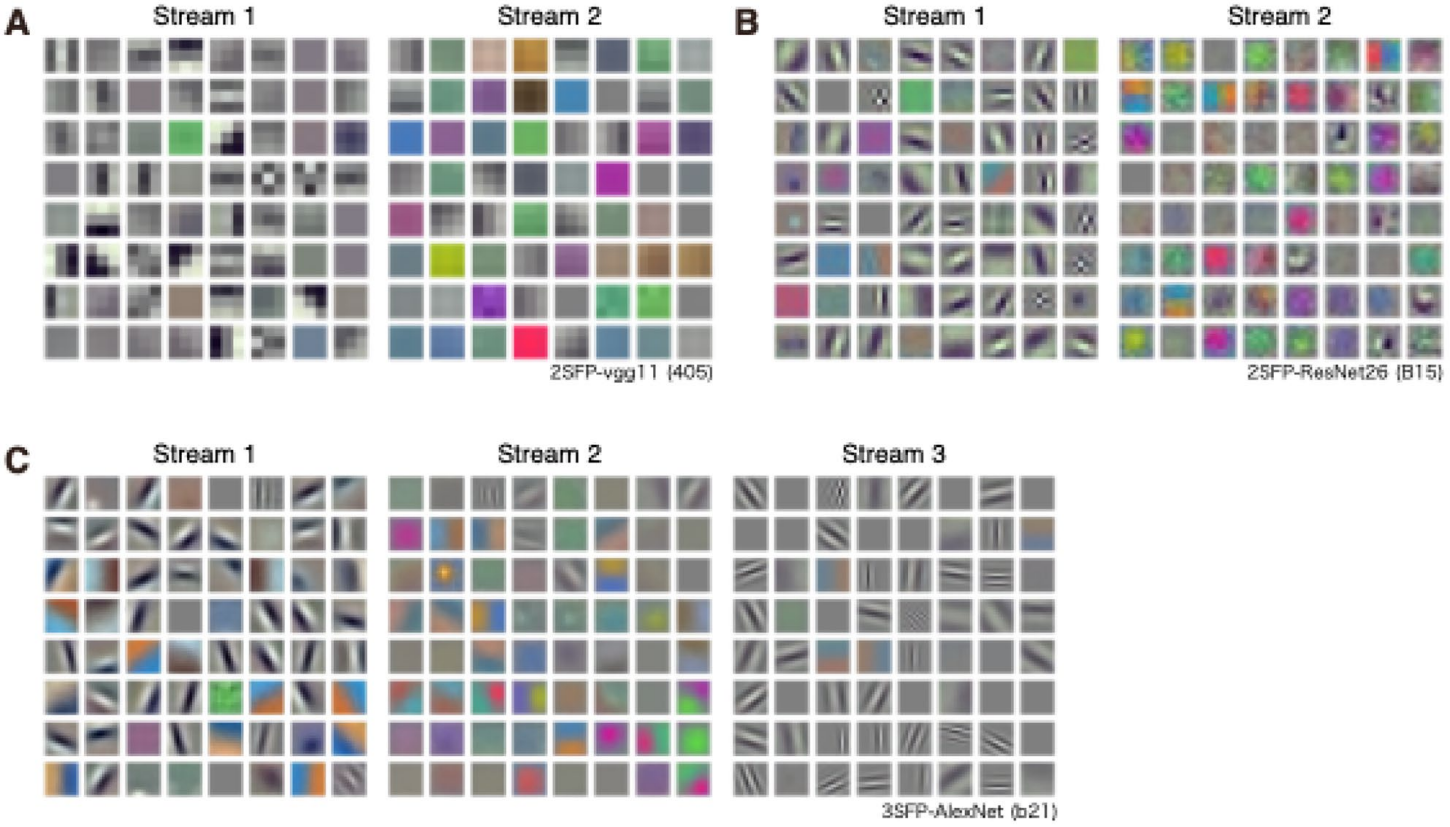
(**A**) Visualization of weight of conv1 filters (n = 64) from stream 1 (left) and stream 2 (right) of the two-streams fully parallel (2SFP) VGG11 instance. (**B**) Visualization of weight of conv1 filters (n = 64) from stream 1 (left) and stream 2 (right) of the 2SFP ResNet26 instance. (**C**) Visualization of weight of conv1 filters (n = 64) from stream 1 (left), stream 2 (center), and stream 3 (right) of the three-streams fully parallel AlexNet instance.

To examine whether the segregation of filter properties between two streams of 2SFP-AlexNet was observed in another type of convolutional neural network, VGG11^38^ was parallelized to construct 2SFP-VGG11. 2SFP-VGG11 has two streams of eight hierarchically organized convolutional layers and five pooling layers. Outputs from each stream were combined and fed into fully connected layers, then to the output layer. Similar to the results obtained with 2SFP-AlexNet, segregation of filters according to their properties was observed in 2SFP-VGG11 (Fig. 9A). The color index of conv1 of stream 1 (0.00082, median) was smaller than that of stream 2 (0.022, median; *p* = 0.0096, Mann–Whitney U test). Orientation selectivity and preferred SF of conv1 filters were not examined quantitively, because of the small size (3 × 3) of conv1 filters of the 2SFP-VGG11. Qualitatively, conv1 filters of stream 1 were mostly orientation selective and preferred higher SF, whereas conv1 filters of stream 2 were mostly less orientation selective and preferred lower SF. The results suggested that segregation of information between two streams was observed in the 2SFP-VGG11.

ResNet26^39^, which has different architecture from AlexNet and VGG11, was also parallelized to construct 2SFP-ResNet26. ResNet26 has conv1 and conv2_x to conv5_x layers with skip connections. Each of the conv2_x to conv5_x layers, contains two sets of three convolutional layers. Thus, ResNet26 has 25 convolutional layers with two pooling layers and one fully connected layer. The convolutional layers and pooling layers were parallelized and outputs from each stream of convolutional layers were combined and fed into the fully connected layer. Segregation of filters according to their properties was observed in 2SFP-ResNet26 (Fig. 9B). Color index, orientation index, and preferred SF of conv1 filters differed among the streams (*p* = 1.13 × 10^−6^–1.11 × 10^−9^, Kruskal–Wallis H test). Conv1 filters of stream 1 were mostly orientation selective (0.73, median orientation index), but color selectivity was low (0.027, median color index), and preferred modest SF (1.0, mean preferred SF). Conv1 filters of stream 2 were mostly color selective (0.43, median color index), but weakly selective to orientation (0.18, median orientation index) and preferred lower SF (0.23, mean preferred SF).

Segregation of functional properties across 3SFP-AlexNet was also examined. Color index, orientation index, and preferred SF of conv1 filters differed among three streams (*p* = 2.99 × 10^−16^–7.67 × 10^−11^, Kruskal–Wallis H test; Fig. 9C). Conv1 filters of stream 1 were mostly orientation selective (0.61, median orientation index), but color selectivity was low (0.018, median color index), and preferred modest SF (0.90, mean preferred SF). Conv1 filters of stream 2 were mostly color selective (0.66, median color index), but weakly selective to orientation (0.25, median orientation index) and preferred lower SF (0.48, mean preferred SF). Conv1 filters of stream 3 were also mostly orientation selective (0.85, median orientation index), but color selectivity was low (0.0021, median color index), and higher SF was preferred (2.31, mean preferred SF). Thus, if there are three streams, a stream contains color-selective and low SF-preferring filters, another stream contains orientation-selective and high SF-preferring filters, and yet another stream contains orientation-selective and modest SF-preferring filters. Similar segregation in multiple streams of parallelized or branched CNNs has been reported previously^40^.

## Discussion

**Figure 10:**
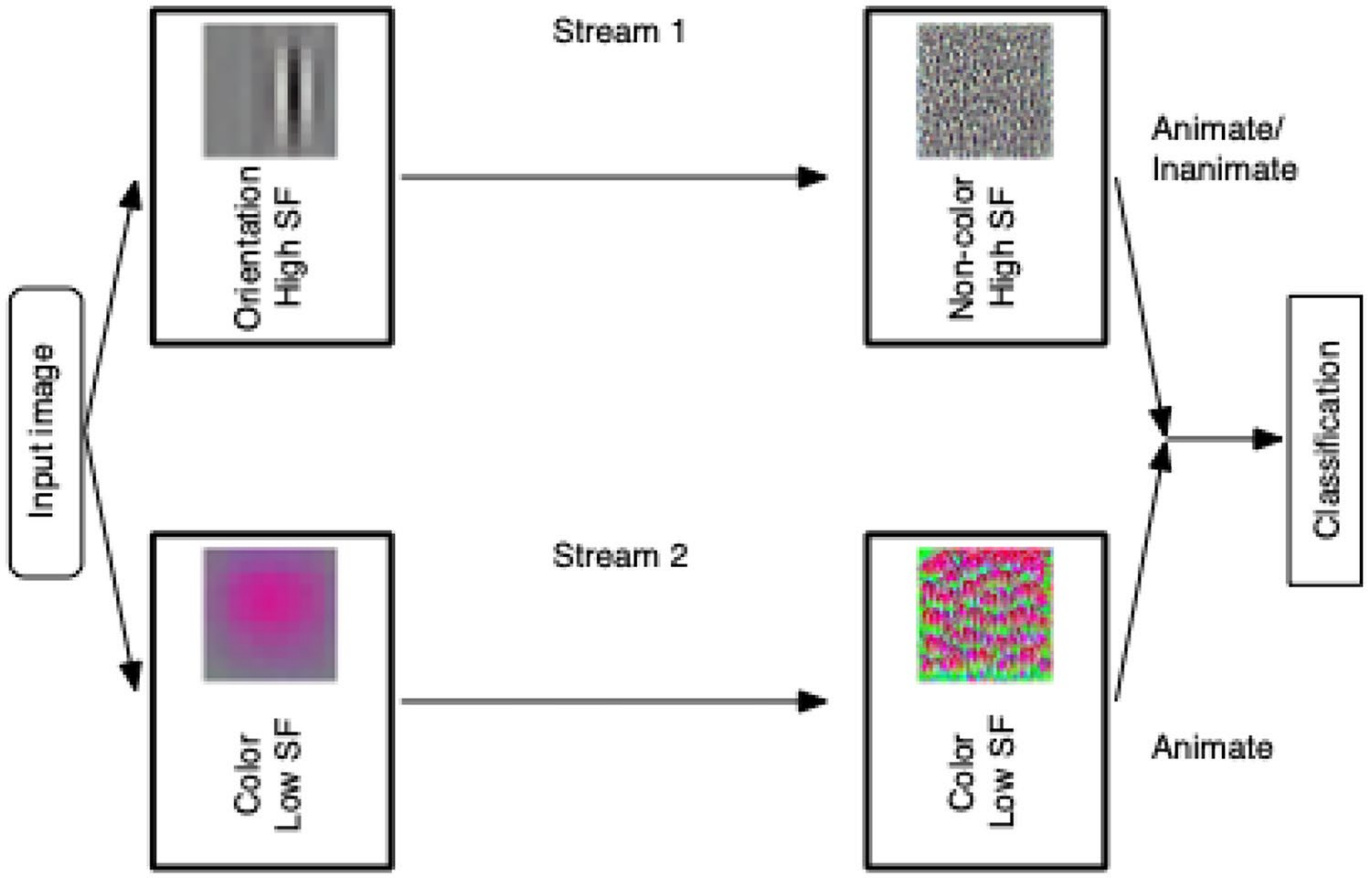
Schematic summary of the present study. Filters in a stream of two-streams fully parallel AlexNet are orientation selective and non-color selective and prefer higher spatial frequency (SF). Filters in the other stream are color selective and prefer lower SF. The color stream contributes to classification of animate images, while the shape stream contributes to classification of both animate and inanimate images.

The main finding of the present study was that color information was segregated from shape information in parallel streams of the CNN, and the color stream was related to classification of animate images (Fig. 10). The results suggest that properties of filters and functions of a stream were spontaneously segregated in parallel streams of the CNN without intentionally assigning a particular property and function to a stream. By implementing and training parallel CNNs, it becomes possible to examine the possibility of spontaneous segregation of visual information in parallel streams.

In the present study, I constructed a modified version of AlexNet (i.e., 2SFP-AlexNet), which has two fully parallelized streams from conv1 to conv5. Previous studies of AlexNet introduced parallel architecture, in conv1 and conv2^27,33^, or other studies introduced parallel architecture using networks that were different from the AlexNet^41–43^. In these studies using AlexNet^27,33^, color-agnostic kernels were spontaneously segregated from color-specific kernels in conv1. In the present study, such a segregation was confirmed. Furthermore, introduction of parallel architecture throughout the convolutional layers allowed deletion of a stream and revealed that deletion of a stream decreased classification performance of animate images.

In the present study, neural networks were trained for classification of 1000 object-image categories using the ImageNet database^35^. These image categories were divided into animate and inanimate super-categories, and the effects of stream-deletion experiments were analyzed using super-categories. The results depended on the image database used for the training. If an image database other than ImageNet is used, such as a scene-image or face-image database, or combinations of these databases, each stream acquires different functions. Analysis using diverse images should be performed to confirm the universality of the present results.

The present study used an early type of CNN (i.e., AlexNet) and confirmed the results with VGG and ResNet. Studies using more recently developed neural networks, such as Swin Transformer^44^ and ConvNeXt^45^, are important. Future studies should investigate whether parallelization of recent networks also induces segregation of filter properties and functions.

Despite the limitations mentioned above, the present results are consistent with previous findings in primate visual cortical areas. For example, most neurons in V1 of the primate brain are reported to be selective to a single visual modality^46^. Color-selective neurons are less orientation selective and tend to prefer lower SF, while orientation-selective neurons are less color selective and prefer higher SF^46^. These properties are consistent with the properties of filters of 2SFP-AlexNet.

The present study also replicated functional organization in primate visual cortical areas.

In lower visual cortical areas of the primate brain, such as V1 and V2, color information and orientation information are processed in different cortical modules^2,6,8–18^. Furthermore, lower SF-preferring neurons are found in a specific compartment, while higher SF-preferring neurons are found in another compartment in V1^47,48^. In the higher visual cortical areas, color information and shape information are processed in a segregated manner^19–22^. Such a segregation of color, orientation, and SF information was observed in the present 2SFP-AlexNet. In the inferior temporal cortex, animate images are processed in a segregated manner from inanimate images^23–26^. The results of the deletion study are consistent with the presence of animate modules.

Although segregation of color information and orientation information in early visual cortical areas is generally accepted^7^, there is substantial variation in the results of physiological studies that examined segregation of color-selective neurons and orientation-selective neurons in compartments revealed by cytochrome oxidase staining^2,8–18^. Interestingly, the findings of Flachot and Gegenfurtner^33^ and the current study also revealed substantial variation in the degree of segregation of color information and shape information among model instances.

Some of the results obtained in the present study have not previously been examined in the primate brain. RDM analysis of the present study revealed that image representation by conv1 filters in the color stream was similar to that in the shape stream. It may be useful for future research to investigate whether image representation in the color compartment is similar to that in the shape compartment of the primate brain. Single stream-deletion experiments revealed an association between color stream and animate image classification. Future studies should be conducted to clarify this relationship in the primate brain.

## Methods

2SFP-AlexNet was constructed and trained using the PyTorch framework (v.1.12.0)^49^. 2SFP-AlexNet contains two streams of five hierarchically organized convolutional layers (conv1–5) and three pooling layers (Fig. 1A). Outputs from the two streams were combined and fed into fully connected layers, then to the output layer. 2SFP-AlexNet was initialized randomly and trained for classification of 1000 object categories using the ImageNet database^35^, which contains 1.2 million training images and 50,000 validation images. The size of images was 224 × 224 pixels. The training was performed using stochastic gradient descent^50^ with cross-entropy loss^51^. The number of epochs was 90. The initial learning rate was 0.01, but was 0.005 or 0.02 in some instances to see the effect of learning rate on the degree of information segregation (Table 1). The learning rate was reduced two times every 30 epochs by 0.1. The momentum was 0.9. The batch size was 128, but 16, 32, or 512 images were tested in some instances to examine the effect of batch size on the degree of information segregation (Table 1).

Color selectivity and orientation selectivity of each filter of the conv1 layer were quantified with selectivity indices. If a filter did not develop any structure (i.e., flat kernel; for example, see the 4th filter in the first row of stream 1 of Fig. 1B-right), the filter was excluded from the analyses of index. Color selectivity was evaluated by calculating the correlation coefficient (*r*) of filter weight among red (R), green (G) and blue (B) channels. If a filter was not color selective, weight values were correlated among channels. The smallest correlation coefficient among the three correlation coefficients (*r*_*min*_) was selected, and the color index was obtained with the following formula:

$${\text{Color index}} = r_{min} \times \left( { - 0.5} \right) + 0.5$$

If kernels of one- or two-color channels were flat, variance was zero and *r* could not be defined. In this case, however, it was obvious that the filter was color selective and color index was set to one. The color index took a value between zero and one, and the larger the color index, the higher the color selectivity. Orientation selectivity was quantified with the following formula after two-dimensional discrete Fourier transform:

$${\text{Orientation index}} = \left( {{\text{Amplitude}}_{{\mathrm{p}}} - {\text{Amplitude}}_{{\mathrm{o}}} } \right)/\left( {{\text{Amplitude}}_{{\mathrm{p}}} + {\text{Amplitude}}_{{\mathrm{o}}} } \right)$$

Here, Amplitude_p_ and Amplitude_o_ are the filter weight amplitude at preferred and orthogonal orientation, respectively. Amplitude was calculated by summating the amplitude within ± 15° and was examined with an interval of 30°. Orientation index was calculated using the preferred color channel, which has the largest weight amplitude. Orientation index takes a value between zero and one, and the larger the orientation index, the higher the orientation selectivity. Preferred spatial frequency (SF, cycles/filter) of each filter of conv1 layer was examined by summating amplitude along the circumference at each frequency using the preferred color channel. Because the size of conv1 filters was 11 × 11, SF was examined from zero (DC) to 5 cycles/filter.

To examine the properties of filters in higher convolutional layers (conv2–5), which have more than three channels and filter weights were difficult to visualize with RGB values, the stimulus image (most effective stimulus, MES) that induced the strongest activation in each filter was calculated using gradient ascent starting from an initial image with random RGB values^36,37^. The mean across all the units that constitute a filter was maximized. The image size was 224 × 224, which was the same as that of the images used in the training and validation sets. Color selectivity of MES was evaluated by calculating the correlation coefficient (*r*) of RGB values among RGB channels. The smallest correlation coefficient among the three correlation coefficients (*r*_*min*_) was selected, and color index values were obtained with the following formula.

$${\text{Color index}} = r_{min} \times \left( { - 0.5} \right) + 0.5$$

Orientation selectivity was not quantified because many of the MESs did not display clear selectivity to orientation. Preferred spatial frequency (SF, cycles/image) of each MES of conv2–5 was examined by summating amplitude along the circumference at each frequency at the preferred color. Because the size of the filters was 224 × 224, SF was examined from zero (DC) to 112 cycles/image.

To compare representation of a set of stimulus images between streams of 2SFP-AlexNet, RDM^24^ was calculated. From each of 1000 categories of the validation set of ImageNet, one stimulus image was randomly selected and a set of 1000 stimulus images was created for RDM analysis. The set was consistently used in the present analysis. Filter outputs were calculated for each stimulus image. For example, in the case of conv1, outputs from 64 × 55 × 55 filters were calculated. Rank ordered and normalized distances between outputs of the set of filters to a pair of stimulus images were then calculated. Once RDM for each convolutional layer was calculated, similarity in stimulus representation was quantified by calculating the correlation coefficient between RDMs.

To examine the contribution of each stream to image classification, a deletion experiment was performed. To delete a stream, output values of the last max-pool layer of the stream were forcibly set to zero or to its average value during the validation trial. The top-1 accuracy (classification accuracy) was calculated for each of 1000 image categories with the validation set. The effects of deletion were examined by calculating the difference in classification accuracy between original 2SFP AlexNet and stream-1 deleted 2SFP AlexNet, and that between original 2SFP AlexNet and stream-2 deleted 2SFP AlexNet (ΔClassification accuracy = accuracy of original 2SFP-AlexNet − accuracy of stream deleted 2SFP-AlexNet). The ΔClassification accuracy was sorted in descending order. Because each image category is labeled in the animate or inanimate super-category, the rank order of animate and inanimate super-categories was obtained. The rank order was replotted in a x–y plane according to the animate and inanimate super-category (see Supplementary Fig. 1). From the graph plotted in an x–y plane, AUC was calculated. AUC was normalized with the product of the number of image categories in the animate super-category (*n* = 398) and that in the inanimate super-category (*n* = 602). AUC takes a value between 0 and 1. AUC is larger than 0.5 if many image categories in the animate super-category showed large decreases in the proportion of correct responses and had higher ranks than those in the inanimate super-category. AUC is smaller than 0.5 if many image categories in the inanimate super-category showed large decreases in the proportion of correct responses and had higher ranks than those in the animate super-category. For AUC values, significance was evaluated using label randomized data (*the number of randomizations* = 1000).

### Statistical analysis

All data were pooled for statistical analyses. Analyses were performed with pandas, numpy, scipy, scikit-learn, and visualized with matplotlib and seaborn on Python. The statistical tests used in the present study were the Mann–Whitney U test (two-tailed), Friedman test for repeated samples, and Kruskal–Wallis H test. Correlation coefficients were examined with Spearman’s rank correlation. The statistical threshold for *p* values was set at 0.01. Median values were calculated to represent a population except for the SF of conv1, in which the median could not capture the difference between groups and the mean value was calculated.

### Supplementary Information

Supplementary Figure 1.

## Data Availability

Parts of the datasets generated during and/or analyzed during the current study are available at the Osaka University Knowledge Archive (https://hdl.handle.net/11094/94838; 10.60574/94838). The remaining part of the data are available from the corresponding author on reasonable request.

## Code availability

Parts of the computer code used during the current study are available at the Osaka University Knowledge Archive (https://hdl.handle.net/11094/94838; 10.60574/94838). The remaining computer code is available from the corresponding author on reasonable request.
