# Supplementary material for: An analysis of information segregation in parallel streams of a multi-stream convolutional neural network
Source: Sci Rep. 2024 Apr 20;14:9097. doi: 10.1038/s41598-024-59930-7 (PMC11032341; doi:10.1038/s41598-024-59930-7)
Supplement: Supplementary file 1 — Supplementary Figure 1. [file 41598_2024_59930_MOESM1_ESM.docx]

Supplementary Figure 1

Method of constructing a two-dimensional (2D) plot (Fig. 8D, E) of the descending rank order of animate and inanimate super-categories from the plot of differences in classification accuracy (𝛥Classification accuracy = accuracy of original two-streams fully parallel (2SFP)-AlexNet − accuracy of stream deleted 2SFP-AlexNet) between original 2SFP-AlexNet and stream deleted 2SFP-AlexNet (Fig. 8C). ***A***, a plot of 𝛥Classification accuracy on the basis of artificial data consisting of 20 image categories in which 10 belong to the animate super-category and the other 10 belong to the inanimate super-category. In this example, 10 image categories in the animate super-category are ranked high and 10 image categories in the inanimate super-category are ranked low after sorting in descending order. ***B***. The 2D plot of the descending order of the animate and inanimate super-categories of ***A***. In the graph, the inanimate and animate super-categories are plotted on the x and y-axis, respectively. Each point is plotted according to the following rule starting from the origin (x = 0, y = 0). If an image category belongs to the animate super-category, the y-value is incremented by one, whereas if an image category belongs to the inanimate super-category, the x-value is incremented by one. In the case of ***A***, the first-ranked image category of the sorted rank order belongs to the animate super-category, the y-value is incremented by one, and the x-value is kept at 0. Similarly, image categories of 2nd to 10th sorted rank order also belong to the animate super-category, the y-value is increased to 10, and the x-value is kept at 0. Thus, the vertical line can be seen at x = 0. The last 10 (11th to 20th) image categories of the sorted rank order belong to the inanimate super-category, the x-value is increased from 0 to 10, and the y-value is kept at 10. Thus, the horizontal line can be seen at y = 10. The area under the curve (AUC) is normalized with the product of the number of image categories in the animate super-category (10), and the number of image categories in the inanimate super-category (10). In this example case, AUC is 1. ***C***, Differences in classification accuracy (𝛥Classification accuracy) on the basis of artificial data consisting of 20 image categories in which 10 belong to the animate super-category and the other 10 belong to the inanimate super-category. In this artificial case, 10 image categories in the inanimate super-category are ranked high, and 10 image categories in the animate super-category are ranked low after sorting in descending order. ***D***. In the case of ***C***, the first 10 image categories of the sorted rank order belong to the inanimate super-category, the x-value is increased from 0 to 10, and the y-value is kept at 0. Thus, the horizontal line can be seen at y = 0. The last 10 image categories of the sorted rank order belong to the animate super-category, the y-value is increased from 0 to 10, and the x-value is kept at 10. Thus, the vertical line can be seen at x = 10. In this case, the normalized AUC is 0. In this way, the AUC quantifies the sorted rank order of the animate and inanimate super-categories in 𝛥Classification accuracy.
